# A systematic pipeline for evaluating factors affecting radar-based microwave breast imaging quality

**DOI:** 10.1088/2057-1976/aea856

**Published:** 2026-10-07

**Authors:** Eleonora Razzicchia, Yunxiao Zhang, Anthony Gracioppo, Ali Farshkaran, Sibi C Shanmugavel, Shwetadwip Chowdhury, Emily Porter

**Affiliations:** 1 Department of Biomedical Engineering, McGill University, Montreal, QC, Canada; 2 Department of Electrical and Computer Engineering, McGill University, Montreal, QC, Canada; 3 Department of Physics, McGill University, Montreal, QC, Canada; 4 Department of Electrical and Computer Engineering, University of Texas at Austin, Austin, TX, United States of America

**Keywords:** antenna systems, breast cancer, microwave imaging, numerical phantoms, radar-based algorithms, radar techniques

## Abstract

Microwave imaging (MWI) has emerged as a promising non-ionizing, cost-effective technique for breast cancer detection. However, the influence of key system design choices remains poorly characterized. This study aims to systematically investigate how antenna array geometry, tumor characteristics, and tissue dielectric properties influence radar-based image reconstruction using a robust computational pipeline. A total of 575 different scenarios were simulated and analyzed, combining 25 distinct breast models with varying tumor configurations and 23 antenna configurations (including 20 randomly selected antenna subsets and 3 uniform arrays). The breast models included one or two tumors with varying radii, embedded in backgrounds with different dielectric properties. Quantitative metrics such as signal-to-noise ratio (SNR), signal-to-mean ratio, localization error, and separation error were used to consistently evaluate the reconstructed images. Additionally, a proof-of-concept experimental study using a laboratory MWI prototype was also conducted to demonstrate the applicability of the proposed pipeline to measured data. Simulation results show that image quality improves with increasing antenna count, while, among arrays with the same number of elements, uniform and symmetric geometries achieve higher average SNR than asymmetric configurations. Tissue relative permittivity and tumor size show limited correlation with the image quality metrics, whereas tumor location within the phantom significantly affects localization accuracy. The experimental proof of concept further demonstrated the capability of the proposed pipeline to compare different types of reconstruction algorithms using the same quantitative image quality metrics. These findings provide quantitative insights into the influence of antenna array design on breast MWI performance and demonstrate the value of systematic simulation-based evaluation frameworks. The proposed methodology can support the optimization of antenna array layouts and facilitate the development of more effective MWI systems prior to clinical translation.

## 1. Introduction

Breast cancer remains one of the most prevalent malignancies and a leading cause of cancer-related mortality among women worldwide (Breast Cancer Research Foundation 2025). Early detection is crucial for improving prognosis and treatment outcomes, highlighting the need for accurate, accessible, and safe screening and diagnostic tools (Coleman 2017). Despite widespread use, conventional imaging modalities such as x-ray mammography, ultrasound, and magnetic resonance imaging (MRI) each present inherent limitations. Mammography, the principal screening technique for breast cancer, involves exposure to ionizing radiation and can be less effective in dense breast tissue (IARC Working Group 2016). As a result, the cumulative low-dose radiation can lead to cancer and, moreover, it is prone to errors, with the potential for false positives, leading to unnecessary anxiety (American Cancer Society 2025). Ultrasound, though non-ionizing and widely available, suffers from limited sensitivity and high operator dependence, leading to variability in interpretation (Tabriz *et al* 2011). MRI offers high-resolution imaging, but its high cost, limited availability, and issues such as patient discomfort and claustrophobia restrict its use as a first-line screening tool (Munn *et al* 2015).

In response to these limitations, microwave imaging (MWI) has emerged as a promising alternative or complementary screening technology for breast cancer detection (Kwon and Lee 2016). MWI is a non-invasive, radiation-free, and cost-effective technique. It uses the dielectric contrast between healthy and malignant breast tissues to generate images for tumor localization and characterization (Chandra *et al* 2017).

Recent advancements in MWI have led to the development of multiple systems that have progressed to clinical pilot studies (Porter and O’Loughlin 2022, Origlia *et al* 2024). These systems vary significantly in terms of antenna types, numbers, spatial configurations, and operating frequencies. For example, the Italian company UBT s.r.l. developed MammoWave, which employs two antennas that rotate azimuthally across 10 transmitting positions (Sani *et al* 2021, Sánchez-Bayuela *et al* 2023, Álvarez Sánchez-bayuela *et al* 2024). At the University of Calgary, Prof. Fear (co-founder of Wave View Imaging) and her team developed a transmission-based measurement system composed of two planar arrays, with a total of 59 transmitting antennas and 240 receiving antennas (Mojabi *et al* 2025). The UK-based Micrima Ltd. developed the MARIA system, a radar-based multistatic device featuring a 60-antenna array fixed within a hemispherical shell filled with a coupling medium (Sidebottom *et al* 2024). The Wavelia system by MVG Industries (France) performs mechanical scanning via a hemispherical multi-probe array that moves vertically in small increments (Fasoula *et al* 2023, 2025).

While this diversity reflects innovation in the field, it also results in inconsistent imaging outcomes and makes it challenging to directly compare system performance. For instance, certain configurations may perform well in non-dense breast tissue but yield suboptimal results in denser tissue (Porter and O’Loughlin 2022), raising the question of which features define an optimal imaging prototype.

A key determinant of performance in MWI systems is the antenna array configuration. Factors such as symmetry, spacing (equal or non-equal), and array geometry significantly affect the spatial resolution and sensitivity of the reconstructed images (Hazarika *et al* 2016). Various antenna array configurations have been proposed and evaluated in numerous studies (Klemm *et al* 2011, Wang *et al* 2015, Bourqui and Fear 2016, Scapaticci *et al* 2018, Mojabi *et al* 2025, Reimer *et al* 2025), each with distinct implications for imaging resolution, coverage, and system complexity (Reimer *et al* 2025). However, antenna array design is only one of several factors that influence imaging performance. Other key design choices, including the antenna design, operating frequency, measurement configuration, and image reconstruction algorithm, also have a significant impact on the quality of the reconstructed images. Despite their importance, many current MWI prototypes have not been systematically evaluated to identify optimal combinations of these design parameters. Moreover, although several studies have proposed methods for image quality assessment and quantification (Nikolova 2017), there is still no set of standardized metrics, and even commonly used ones often vary in their definitions.

Consequently, we argue that gaining a thorough understanding of these factors in simplified imaging scenarios is essential to inform the design of more complex and clinically viable 3D imaging prototypes. Therefore, this study introduces a robust imaging pipeline designed to serve as an automated tool for the quantitative analysis of MWI-reconstructed images. The proposed pipeline provides a reproducible methodology for objectively assessing a broad range of MWI system design choices under controlled conditions using consistent quantitative image quality metrics. The resulting performance trends provide a comparative benchmark for alternative system designs and establish a foundation for incorporating more comprehensive statistical analyses as larger datasets become available. In this work, the primary focus is on the systematic simulation-based evaluation of antenna array configurations and antenna count across diverse breast models. However, to further demonstrate the versatility of the proposed pipeline, a proof-of-concept experimental study using a cylindrical breast phantom is also presented. Although intended solely as a preliminary validation of the pipeline, this experimental investigation illustrates that the same methodology can be applied to experimentally acquired data and extended to objectively compare other design choices, such as different types of reconstruction algorithms, using the same quantitative evaluation metrics.

Ultimately, unlike existing studies, the proposed pipeline provides a reproducible methodology for objectively assessing the impact of different design choices under controlled conditions, thereby serving as a necessary step toward the development of future microwave breast imaging systems.

## 2. Methods

To investigate the impact of system design choices on MWI performance, we implemented a comprehensive simulation and image reconstruction pipeline. Electromagnetic (EM) simulations were conducted using the high frequency structural simulator (HFSS), which enables accurate modeling of wave interactions within tissues. These simulations were run on Compute Canada’s supercomputing infrastructure to ensure computational efficiency and scalability. To further demonstrate the applicability of the proposed pipeline, controlled experimental measurements were performed as a proof-of-concept using our laboratory MWI prototype (section 2.5).

The resulting *S*-parameter data from both the EM simulations and the experimental measurements were post-processed in MATLAB, where MERIT (microwave radar-based imaging toolbox) (O’Loughlin *et al* 2018) was employed for image reconstruction. Two-dimensional (2-D) reconstructed images were subsequently subjected to quantitative analysis using four image quality metrics: signal-to-noise ratio (SNR), signal-to-mean ratio (SMR), localization error, and separation error. These metrics were consistently applied to enable objective performance comparisons across different scenarios.

### 2.1. Numerical phantom models

A series of simplified cylindrical breast phantoms were employed in this study to strike a balance between anatomical realism and computational feasibility. Although these numerical models do not reproduce the full anatomical complexity of the human breast, their simplified geometry was intentionally adopted to enable controlled experiments and the systematic evaluation of key imaging parameters. This approach facilitates the isolation of specific physical and algorithmic effects while ensuring methodological robustness, reproducibility, and computational efficiency. It is important to note that, despite the simplified phantom geometry, the EM problem remains inherently 3D; therefore, full-wave 3D EM simulations were performed to accurately model EM wave propagation and scattering within the imaging domain, while reconstruction performance was assessed on a representative 2D slice extracted from the reconstructed 3D volume solely for visualization and quantitative analysis.

All phantoms consisted of three main components: an outer skin layer, a homogeneous background tissue, and one or two embedded tumor inclusions (or ‘targets’). A full phantom had a radius of 75 mm and a height of 80 mm. The skin layer was modeled as a 2 mm thick shell using the frequency-dependent dielectric datasets available in the IT’IS Foundation database (IT’IS Foundation 2024), which is primarily based on the dielectric measurements and Cole–Cole models reported by Gabriel *et al* (1996). The background tissue was designed to mimic fatty tissue with varying proportions of glandular content. The dielectric properties of the fatty and glandular tissues were also assigned from the same datasets, with the corresponding relative permittivity and conductivity values defined over the 1–5 GHz operating frequency range.

Embedded tumors were modeled as cylindrical inclusions with radii ranging from 4.5 mm to 7.5 mm. Each inclusion extended through the full height of the phantom (80 mm). These inclusions were characterized by constant dielectric properties at 3 GHz, specifically a relative permittivity of 59 and an electrical conductivity of 1.2 S m^−1^, values representative of malignant breast tissue (Campbell and Land 1992).

For each simulation, the proportion of glandular tissue in the homogeneous background was randomly varied between 0% and 60%. The dielectric properties of the background (relative permittivity and conductivity) were then calculated as a weighted average of glandular and fatty tissue properties according to this proportion, ensuring realistic breast tissue characteristics (Nelson *et al* 2008). Tumor sizes were systematically varied in 0.5 mm increments between 4.5 mm and 7.5 mm, allowing the evaluation of the system’s ability to detect tumors smaller than the clinically relevant lesions (Kranold *et al* 2021). Tumor positions were assigned using random 2D coordinates within a circular region bounded by [−38 mm, 38 mm] in both the *x* and *y* directions. The region for random target placement was defined to allow inclusions to be distributed throughout the interior of the domain while avoiding locations too close to the boundaries, thereby minimizing edge effects and ensuring stable reconstruction conditions. This choice is consistent with clinical evidence, as most breast cancers originate in the glandular tissue (ductal or lobular epithelium), which is typically located in the central and mid-depth regions of the breast (Menon *et al* 2025). The tumor models included both single and two-inclusion scenarios. When two tumors were present, a minimum separation distance was enforced to prevent spatial overlap. This experimental setup enables a robust assessment of MWI’s sensitivity to tumor characteristics commonly encountered in clinical contexts. Lesions smaller than 1 cm are generally classified as stage 0 or stage IA breast cancer, which are associated with five-year survival rates exceeding 99% (Breast Cancer Research Foundation 2025). By targeting tumors with a radius as small as 4.5 mm, the study aims to explore the potential of MWI for detecting subclinical and otherwise undetectable lesions, thereby advancing the capabilities of non-invasive breast cancer detection.

The 25 different breast models are summarized in table 1. As illustrated in figures 1(a) and (b), these cases encompass a broad range of fat-to-glandular tissue distributions, which in turn results in a wide variability in dielectric properties. The histograms in figure 1(b) report the relative permittivity values at 3 GHz, the central operating frequency of the sensing element, at which the relative permittivity of the background (${\varepsilon _{\mathrm{b}}}$) is extracted and subsequently provided as input to the imaging algorithm. As mentioned previously, however, all EM simulations were performed using the frequency-dependent complex dielectric properties of the tissues, such that both the relative permittivity and conductivity varied consistently according to the dielectric datasets available in the IT’IS Foundation database (IT’IS Foundation 2024). Additionally, as shown in figure 2(b), tumor locations are distributed throughout the breast volume, and tumor radii vary across the simulated cases. This variability ensures that the simulation dataset effectively captures a diverse set of clinically relevant conditions, enabling a robust performance assessment of the imaging system.

**Figure 1.**
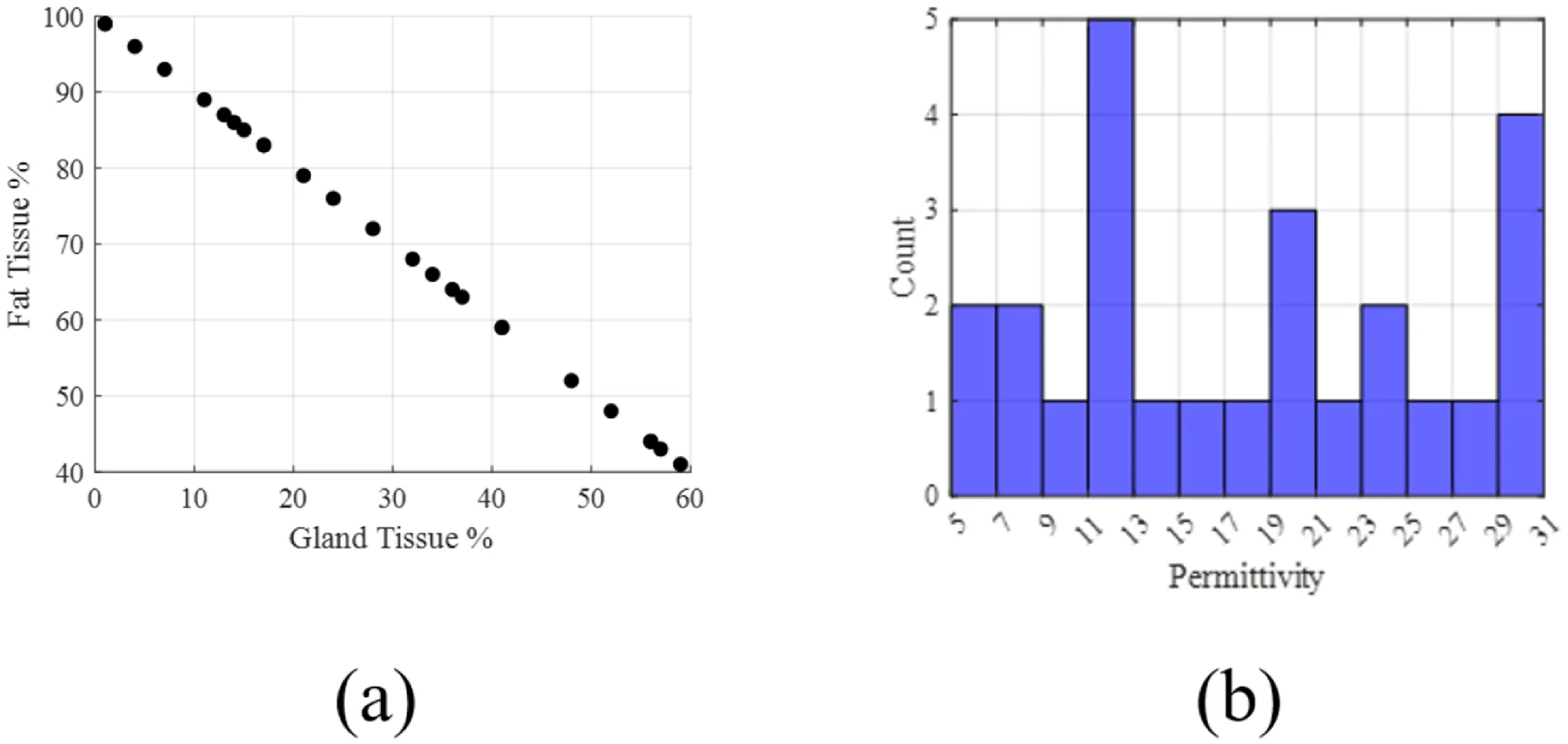
(a) Scatter plot of the fat-to-glandular tissue distribution across the 25 simulation cases. Each point represents a phantom scenario with specific percentages of glandular (*x*-axis) and fat (*y*-axis) tissue, illustrating the wide variability in tissue composition modeled for the study. (b) Histograms showing the distribution of background relative permittivity (${\varepsilon _{\mathrm{b}}}$) values at 3 GHz across all 25 simulation cases.

**Figure 2.**
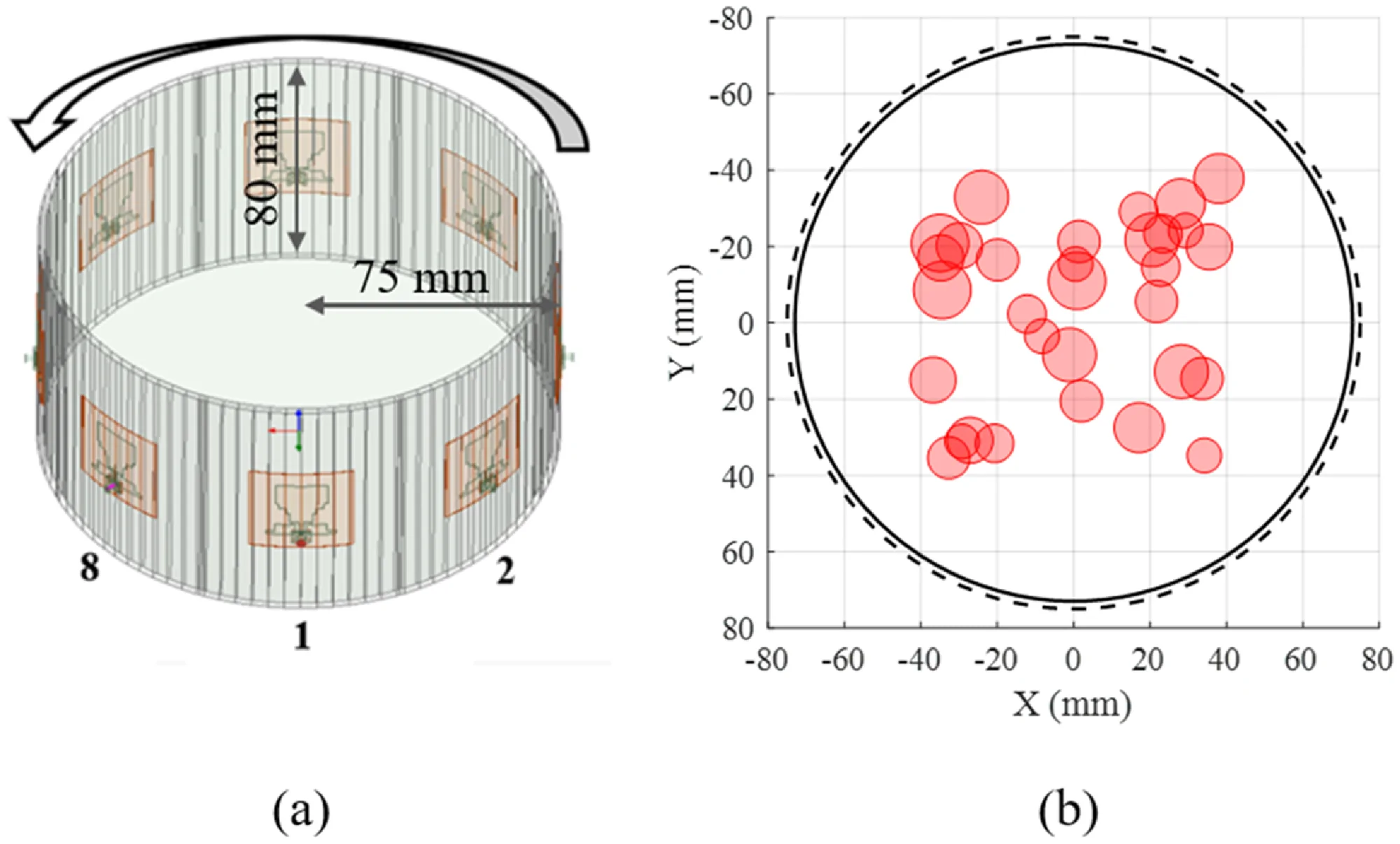
(a) Example of a simplified numerical phantom model implemented in HFSS, shown without tumor inclusions. (b) Spatial distribution of tumor locations and variation in tumor radii across the simulated breast volume. Tumors are randomly positioned within the phantom to ensure a comprehensive assessment of imaging system sensitivity to tumor size and location. The solid circle represents the boundary of the background tissue, while the dashed circle indicates the outer breast surface; the region between them corresponds to the 2 mm skin layer.

**Table 1.** Summary of the 25 breast models, including the number of cases, number of tumors, tumor radius, tumor location coordinates, and inter-lesion separation distances for two-tumor cases. These scenarios represent a diverse range of tissue compositions and tumor characteristics to evaluate imaging performance across varied conditions and lesion separations.

Case #	Gland tissue (%)	Fat tissue (%)	Tumor #	Radius of tumor #1 (mm)	Radius of tumor #2 (mm)	Coordinates of tumor #1 in mm (*X*)	Coordinates of tumor #2 in mm (*X*)	Coordinates of tumor #1 in mm (*Y*)	Coordinates of tumor #2 in mm (*Y*)	Distance between tumors (mm)
1	7	93	1	5.5	—	1.4	—	−21.3	—	—
2	13	87	1	5	—	17	—	−29.1	—	—
3	17	83	1	5.5	—	21.7	—	−5.6	—	—
4	48	52	1	7.5	—	0.9	—	−11	—	—
5	56	44	2	4.5	6	29.3	−27.1	−24.2	30.8	78.8
6	14	86	1	5.5	—	33.6	—	14.6	—	—
7	28	72	1	7	—	20.5	—	−21.7	—	—
8	4	96	2	4.5	7.5	34.2	−35	34.8	−20.9	88.8
9	41	59	1	5	—	22.8	—	−14.6	—	—
10	1	99	2	4.5	6	−29.2	35.5	31.1	−19.9	82.4
11	37	63	1	6.5	—	27.9	—	−31.2	—	—
12	52	48	1	6	—	−29.9	—	−20.2	—	—
13	24	76	2	5.5	7	−32.7	−24.1	35.4	−32.9	68.83
14	15	85	1	7	—	28.2	—	12.8	—	—
15	57	43	2	6.5	6	17.1	−34.8	27.5	−17	68.36
16	41	59	1	4.5	—	0.5	—	−15.4	—	—
17	17	83	1	7.5	—	−34.4	—	−8.6	—	—
18	1	99	2	5	5	−20.7	23.4	31.6	−23.3	70.41
19	59	41	1	5.5	—	−19.9	—	−16.5	—	—
20	34	66	2	6.5	6	38	−36.8	−37.8	15	91.56
21	32	68	1	5	—	−12.2	—	−2.3	—	—
22	11	89	1	4.5	—	−8.3	—	3.5	—	—
23	21	79	2	7.5	5	−1.1	2	−36.9	37.1	74.06
24	59	41	1	5.5	—	7.2	—	8.4	—	—
25	36	61	1	7.5	—	13.1	—	20.5	—	—

All simulations were performed using ANSYS HFSS, which employs a finite element formulation in the frequency domain. This approach enables accurate full-wave modeling of complex antenna geometries, boundary conditions, and dispersive biological tissues, while allowing efficient broadband analysis across the operational frequency range considered in this study. The analysis covered the 1–5 GHz range in order to fully cover the operational bandwidth of the adopted antennas, with data acquired at 81 discrete frequency points to capture the broadband dielectric response of the tissue components. Meshing was carried out using the automatic mesh method. The selected number of frequency samples provides sufficient spectral resolution while maintaining a reasonable computational cost. The simulations were executed on the Alliance supercomputing cluster, with runs submitted in parallel. When server resources were available, all 25 configurations could be completed concurrently, requiring approximately 3 h in total.

### 2.2. Antenna and array configurations

The antenna used in this work is the flexible stepped monopole antenna reported in Bahramiabarghouei *et al* (2015), where details on the design, dimensions, and electrical characteristics are also available. This antenna was selected because it was specifically developed for microwave breast imaging and optimized to achieve wideband impedance matching when operating in direct contact with breast tissue. As mentioned in the previous section, simulations were conducted over the 1–5 GHz frequency range, where 3 GHz is the center operating frequency of the antenna used as the sensing element. The antennas were arranged radially around the breast phantom and excited via a 50 Ω lumped port defined at the feeds, representing a single-tone sinusoidal waveform applied at each analysis frequency.

To evaluate the influence of array configuration on MWI performance, multiple array layouts were investigated. Circular arrays consisting of 12, 8, and 6 equally spaced antennas were simulated using a multi-static acquisition scheme. In this configuration, the full set of transmit–receive antenna combinations was considered, and image reconstruction was performed using the complete multi-static scattering matrix, including both reflection (${S_{ii}}$) and transmission (${S_{ij}}$, i≠j) measurements, to study the effect of element count on image quality and to investigate how progressively reducing the number of antenna elements affects image quality while decreasing hardware complexity. The 12-element array was selected because it represents a practical upper limit on the number of antennas that can be accommodated around the breast while maintaining adequate inter-element spacing. In addition to these uniformly distributed arrays, a series of randomly selected 8-antenna subsets were derived from the original 12-element configuration to explore the role of spatial arrangement. These subsets included both symmetric and asymmetric layouts, with ten unique configurations generated for each category. Representative examples of asymmetric and symmetric arrays are illustrated in figure 3. It is important to note that, although these subsets were randomly selected, resulting in 20 different layouts, to ensure meaningful image reconstruction, the configurations were constrained to guarantee adequate spatial coverage of the imaging domain, as sufficient angular sampling and illumination are required in MWI to provide signal diversity and avoid poorly sampled regions (Razzicchia *et al* 2026). Therefore, layouts with highly clustered elements (e.g. eight antennas placed only on one side of the breast phantom) were not considered.

**Figure 3.**
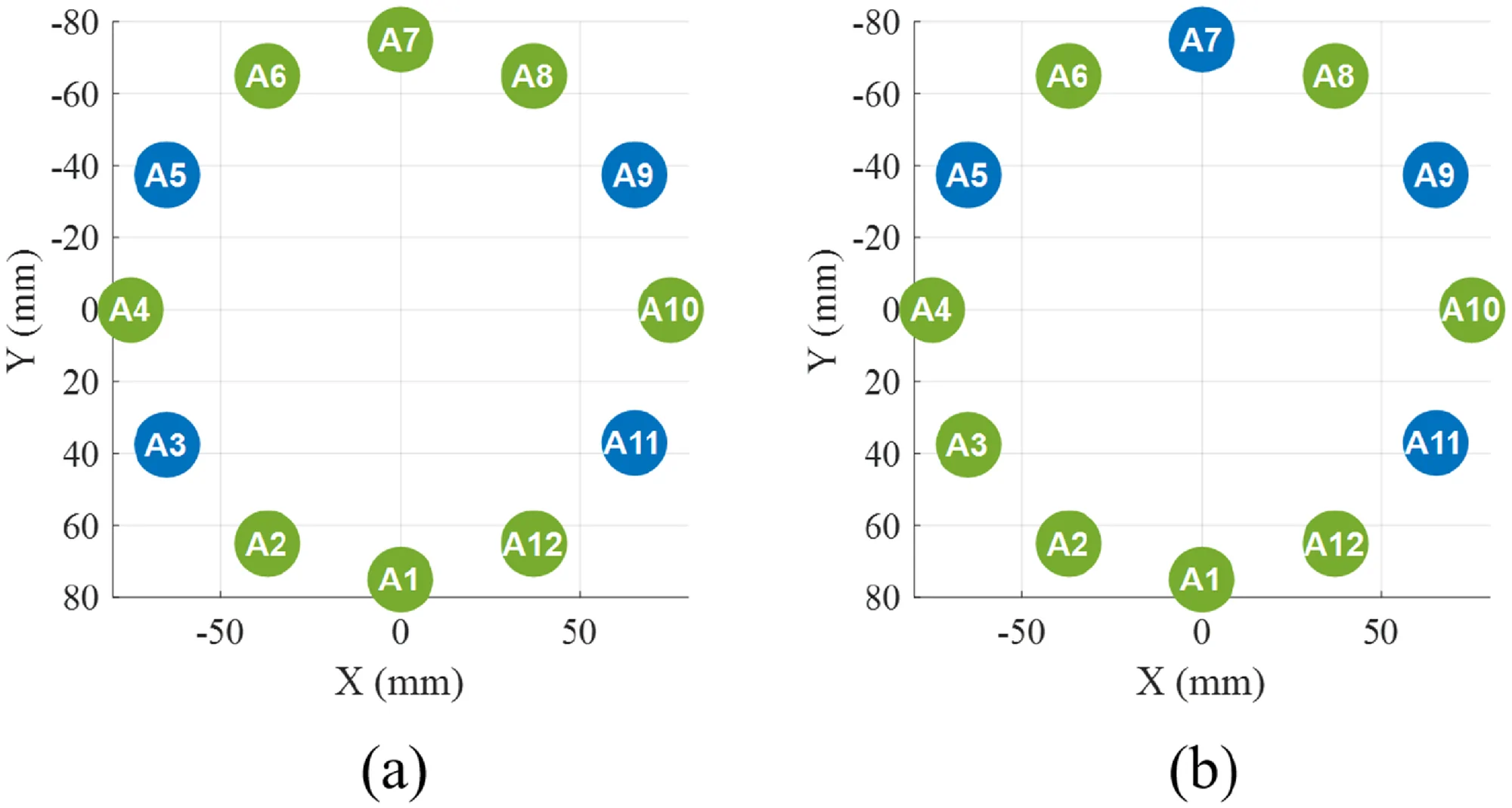
Representative examples of symmetric (a) and asymmetric (b) 8-antenna array configurations derived from the 12-element circular setup. Green circles indicate the excited antennas, while blue circles mark the non-excited antennas. Antenna positions are shown within the breast contour for visualization purposes only.

### 2.3. Image reconstruction algorithm

Image reconstruction was performed using MERIT (O’Loughlin *et al* 2018), an open-source microwave radar imaging toolbox specifically designed for processing radar-based MWI data. MERIT was selected for this study because of its simplicity, robustness, and suitability for handling multi-static data, making it well-adapted for evaluating a large number of simulation scenarios in a consistent manner. In addition, its reconstruction algorithms are openly available, which facilitates reproducibility of the reconstruction pipeline and ensures that the results can be independently verified. For the simulation study, the delay-and-sum (DAS) reconstruction algorithm implemented in MERIT was used. As with other DAS-based approaches, this algorithm has limitations when dealing with complex multiple-scattering environments or when high quantitative accuracy is required. In such cases, model-based algorithms may achieve improved localization and quantification at the expense of higher computational cost. Importantly, the procedures proposed in this work can readily be applied to images generated using any reconstruction algorithm.

The MERIT DAS algorithm reconstructs spatial maps of dielectric contrast by processing backscattered signals collected across multiple transmission and reception channels. These signals, acquired either in the time or frequency domain, are coherently combined to generate an energy distribution that emphasizes regions of significant permittivity variation which are indicative of potential anomalies such as malignant tissue. The algorithm operates on frequency-dependent, complex-valued scattering parameters (*S*-parameters) obtained from EM simulations performed for both tumor-present (‘with target’) and tumor-absent (‘no target’) scenarios. As part of the preprocessing, the tumor-free (reference) case is subtracted from the tumor-present case. This step is standard practice in MWI and serves to remove background contributions as well as system responses not related to the target.

2D reconstructions were extracted at the 50 mm axial slice, chosen as a consistent reference for evaluation. This slice is located slightly above the geometric center of the phantom, corresponding to the central region of the antenna, and captures the area of strongest EM field interaction. We note that the selected axial slice was chosen based on the geometry of the configuration to provide a representative visualization of the reconstruction results. Any 2D slice of the reconstructed volume can be examined for visualization and metric evaluation; therefore, the slice selection affects only the presentation of the results and does not influence the reconstruction process itself. For each simulation case, the initial estimate of the bulk (or effective) relative permittivity (${\varepsilon _{{\mathrm{avg}}}}$) used in the reconstruction algorithm was computed as a volume-weighted average of the skin and background tissue contributions. Specifically, the estimate was given by (1):

\begin{equation*}{\varepsilon _{\mathrm{avg}}} = {V_s} \times {\varepsilon _s}\, + {\text{ }}{V_b} \times {\varepsilon _b},\end{equation*}

where ${V_s} = 5.26\% {\text{ }}$ is the volume fraction occupied by the skin, ${\varepsilon _{\mathrm{s}}}$ ≈37 is the relative permittivity of the skin, ${V_{\mathrm{b}}} = 94.74\% $ is the volume fraction of the background tissue, and ${\varepsilon _{\mathrm{b}}}$ is the relative permittivity of the background medium, which varies according to the simulated glandular content. This approach provides a consistent starting point for all reconstructions and accounts for heterogeneous contributions from the different tissue layers. An example of image reconstructions for all cases using data collected with the uniform 12-antenna array is shown in figure 4.

**Figure 4.**
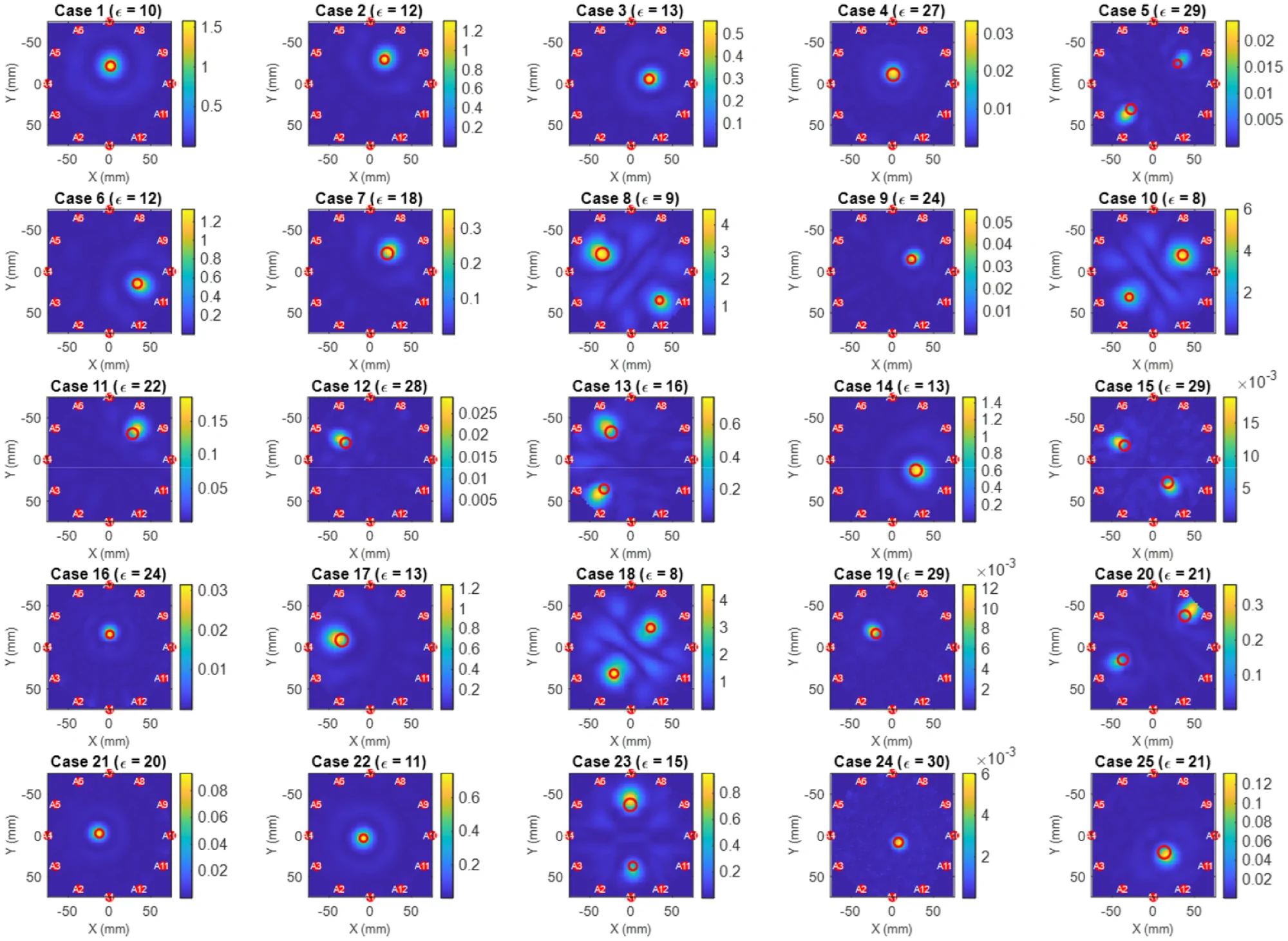
Image reconstructions for all cases using data from a 12-element uniform antenna array with frequencies ranging from 1–5 GHz. The MERIT DAS algorithm was applied with the guessed permittivity set to the estimate of the bulk permittivity (${\varepsilon _{{\mathrm{avg}}}}$). The ‘no target’ cases, in which the phantom contained no tumor inclusions, were used as baseline references during the image reconstruction process. Specifically, the data collected from these control simulations were subtracted from their corresponding ‘with target’ cases to isolate tumor-induced signal changes. Red circles indicate the actual tumor locations, while red dots with labels mark the antenna positions. The reconstructed images are presented on a non-normalized intensity scale, as the emphasis is on relative contrast across scenarios. Cases with lower background relative permittivity generally exhibit higher intensities.

### 2.4. Quantitative image analysis

Reconstructed images were evaluated using a set of objective quantitative metrics designed to assess image quality, detection accuracy, and spatial resolution. As introduced earlier in the text, these metrics include the SNR, SMR, tumor localization error, and tumor separation error (for multi-target cases). This set of metrics enables a comprehensive evaluation of the imaging system performance across different array configurations and tissue heterogeneity scenarios. In particular, SNR in this work quantifies the strength of the tumor response relative to the noise floor (i.e. background artifact level), while SMR measures its contrast relative to the mean background level. Higher values of these metrics generally correspond to clearer tumor signatures and more reliable detectability, whereas lower values indicate a greater risk that tumors may not be distinguished from background responses (i.e. false negatives). Thus, the reported SNR and SMR should be interpreted as contrast-based metrics quantifying the relative intensity between target-dominated regions and background contributions (including clutter-related responses) within the reconstructed image, rather than as measures of noise in the conventional sense.

As an internal validation step, all metric definitions were first tested on ideal phantom images (i.e. MATLAB-generated ground-truth distributions with tumors at their exact positions). In this case, localization and separation errors reduce to zero and the SNR is arbitrarily high, confirming the correctness of the metric implementation before applying them to reconstructed images. The implementation of the metrics is described as follows.

1)**SNR:**

The SNR was computed by adaptively selecting a threshold intensity value for the representative image slice (50 mm axial slice) in each acquisition scenario. For each scenario, the image slice was denoted as ${I_i}\left( {x,y} \right)$, where the index i identifies a specific configuration under analysis, defined by the number and positions of the antennas, as well as the acquisition settings (e.g. different background properties and tumor locations and sizes). A threshold ${T_i}$ was selected for each slice based on a specific percentile of its pixel intensity distribution. Pixels with intensities above this threshold were classified as signal, while those below or equal to the threshold were considered noise.

The mean squared intensity (i.e. power) was computed separately for the signal and noise regions, and the SNR for slice i was defined as the ratio of these powers, expressed in decibels (dB), as shown in equation (2):

\begin{equation*}{\mathrm{SNR}}\left( i \right){\text{ }} = 10{\text{ lo}}{{\mathrm{g}}_{10}}\left( {\frac{{{\mathrm{M}}{{\mathrm{S}}_{\mathrm{s}}}\left( i \right)}}{{{\mathrm{max}}\left( {{\mathrm{M}}{{\mathrm{S}}_{\mathrm{n}}}\left( i \right),\alpha } \right)}}} \right)\end{equation*}

where α is a small positive constant used to ensure numerical stability and ${\mathrm{M}}{{\mathrm{S}}_{\mathrm{s}}}(i$) and ${\mathrm{M}}{{\mathrm{S}}_{\mathrm{n}}}(i$) ) represent the intensities computed over the signal region (tumor area) and the background/noise region, respectively, for the $i{\mathrm{th}}$ slice. These are defined in (3) and (4):

\begin{equation*}{\mathrm{M}}{{\mathrm{S}}_{\mathrm{s}}}\left( i \right) = \frac{{{\mathrm{su}}{{\mathrm{m}}_{\mathrm{s}}}\left( i \right)}}{{{\mathrm{max}}\left(\left| {{S_i}} \right|,1\right)}}\end{equation*}

\begin{equation*}{\mathrm{M}}{{\mathrm{S}}_{\mathrm{n}}}\left( i \right) = \frac{{{\mathrm{su}}{{\mathrm{m}}_{\mathrm{n}}}\left( i \right)}}{{\max \left( {|{N_i}{\mathrm{|}},1} \right)}}{\text{ }}.\end{equation*}

Here, $\mid {S_i}\mid $ and $\mid {N_i}\mid $ are the number of signal and noise pixels, and ${\mathrm{su}}{{\mathrm{m}}_{\mathrm{s}}}\left( i \right)$ and ${\mathrm{su}}{{\mathrm{m}}_{\mathrm{n}}}\left( i \right)$ are the corresponding sums of squared intensities, as shown in (5) and (6):

\begin{equation*}{\mathrm{su}}{\mathrm{m}_{\mathrm{s}}}\left( i \right) = {\text{ }}\mathop {\mathop \sum \nolimits }\limits_{k \in {S_i}} {k^2}\end{equation*}

\begin{equation*}{\mathrm{su}}{\mathrm{m}_{\mathrm{n}}}\left( i \right) = {\text{ }}\mathop {\mathop \sum \nolimits }\limits_{k \in {N_i}} {k^2}.\end{equation*}

The sets ${S_i}$ and ${N_i}$ are defined as in (7) and (8), with k being the intensity value of a pixel in the image:

\begin{equation*}{S_i} = \{k \in {S_i}{\text{ | }}k &gt; {T_i}{\text{ }}\} \end{equation*}

\begin{equation*}{N_i} = \{k \in {N_i}{\text{ | }}k &lt; {T_i}{\text{ }}\} .\end{equation*}

The threshold (${T_i}$) used to distinguish between signal and noise is defined in (9). This was determined using the dataset with the highest number of sensors and was set adaptively by sweeping through percentiles (i.e. 80%–99%) to identify the value that maximizes the average SNR across all images (see figure 5). The final threshold was set at the 94th percentile of the pixel intensity distribution for the slice ${I_i}(x,y$):

\begin{equation*}{T_i}{\text{ = }}\,{\mathrm{percentil}}{\mathrm{e}_{94}}\left( {{I_i}} \right)\end{equation*}

**Figure 5.**
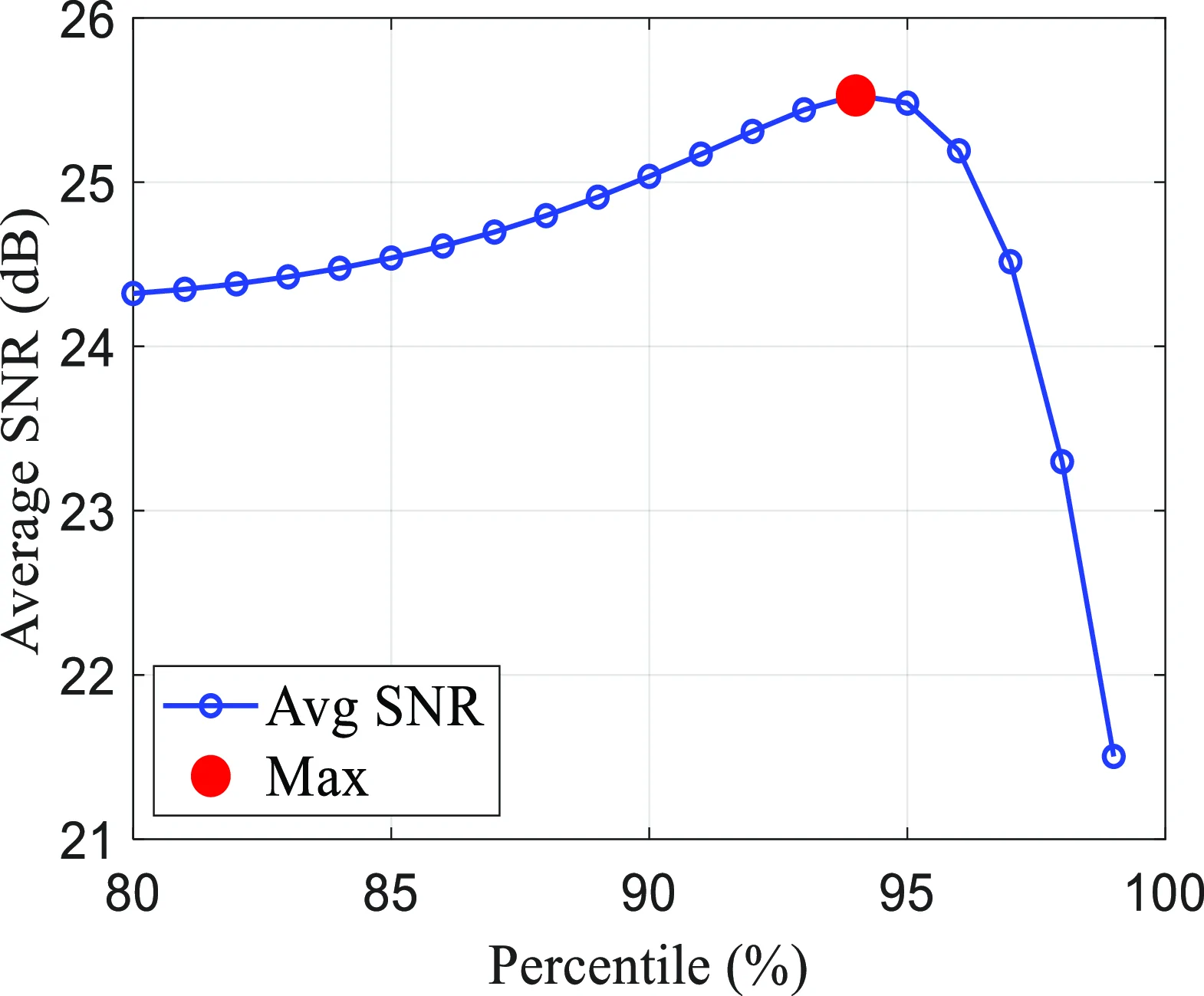
Adaptive threshold selection for SNR optimization. The *x*-axis shows threshold percentiles swept from 80% to 99%, while the *y*-axis displays the corresponding average SNR computed over all cases. The optimal threshold maximizing SNR is identified at the 94th percentile, which was used as the final intensity cutoff for signal classification.

This adaptive approach tailors the threshold to the underlying image data, providing a more robust classification than an arbitrarily fixed threshold. The threshold was selected from the 12-sensor case because the higher SNR yielded a more reliable intensity distribution with better tumor–background separation, ensuring a stable estimate. In this case, the SNR–percentile curve had a maximum at 94%. Scenarios with fewer sensors exhibited higher maxima; for example, the 8-sensor case peaked near 98% due to weaker tumor–background separation. However, the 98% threshold lies in the extreme tail of the distribution, where small percentile changes cause large swings in SNR and thus lead to instability. By contrast, the 94% threshold falls within a plateau region, providing nearly maximal SNR with much lower sensitivity to percentile choice. For this reason, the 94th percentile was adopted as a robust working point across all scenarios. It is important to note that, in this work, the threshold was selected according to a specific criterion in order to simplify the analysis and maintain the focus on the antenna array design aspects. However, metrics such as SNR and SMR can vary as a function of signal-to-background differentiation approaches and threshold levels (Ramos Flores *et al* 2026). Furthermore, we acknowledge that, in practical systems, the threshold should be defined based on system-level parameters (e.g. noise floor or dynamic range).

As an additional quantitative analysis, statistical significance was assessed using the SNR values calculated from the reconstructed images of the 25 simulated breast phantoms. Since each phantom was reconstructed using all antenna configurations, the resulting SNR values were treated as paired data. Two predefined comparisons were considered: (i) symmetric versus asymmetric 8-element arrays and (ii) uniform 8-element versus uniform 12-element arrays. For the first comparison, the SNR values obtained from the ten symmetric configurations were averaged separately for each phantom, and the same procedure was applied to the ten asymmetric configurations, yielding one paired mean SNR value per phantom for each array type. For the second comparison, the SNR values obtained with the uniform 8-element and 12-element arrays were paired directly for each phantom, as it provides a robust non-parametric method for assessing paired differences. Statistical significance was assessed using a two-sided Wilcoxon signed-rank test. A significance level of p<0.05 was adopted.

2) **SMR:**

Using the same adaptive thresholding approach, SMR was calculated as in (10), as the ratio of the mean squared intensity in the signal region to that of the entire image:

\begin{equation*}{\mathrm{SMR}}\left( i \right) = 10{\text{ lo}}{{\mathrm{g}}_{10}}\left( {\frac{{{\mathrm{M}}{{\mathrm{S}}_{\mathrm{s}}}\left( i \right)}}{{{\mathrm{max}}\left( {{\mathrm{M}}{{\mathrm{S}}_{{\mathrm{all}}}}\left( i \right),\alpha } \right)}}} \right)\end{equation*}

where MS_all_(i) is defined in (11) as:

\begin{equation*}{\mathrm{M}}{{\mathrm{S}}_{{\mathrm{all}}}}\left( i \right) = \frac{{{\mathrm{su}}{{\mathrm{m}}_{\mathrm{s}}}\left( i \right) + {\text{ su}}{{\mathrm{m}}_{\mathrm{n}}}\left( i \right)}}{{\max {\mathrm{(}}\left| {{S_i}{\mathrm{|}} + } \right|{N_i}|,1)}}\end{equation*}

**3) Localization error:**

To assess spatial accuracy, a binary mask was generated by applying the threshold to each reconstructed image, isolating high-intensity regions. Centroids of these regions were computed using region-based analysis, then mapped to physical coordinates. The location error was defined as the Euclidean distance (in millimeters) between each centroid and the true center of the corresponding tumor, as defined in (12):

\begin{align*}{\text{Localization Error}} = \| {c_{{\mathrm{reconstucted}}}} - {\text{ }}{c_{{\mathrm{true}}}} \| \end{align*}

**4) Separation error:**

For cases with two tumors, separation error quantified how accurately the reconstruction preserved the spatial separation between inclusions. This was computed as the absolute difference between the reconstructed and true inter-centroid distances, as shown in (13):

\begin{align*}{\text{Separation Error}} = | {d_{{\mathrm{reconstucted}}}} - {\text{ }}{d_{{\mathrm{true}}}} |.\end{align*}

### 2.5. Experimental system setup for pipeline demonstration

To provide an initial experimental assessment of the proposed pipeline, a proof-of-concept study was conducted using a cylindrical breast phantom. The objective of this initial experiment was not to reproduce all the simulation scenarios investigated in this work, but rather to demonstrate the applicability of our framework under controlled experimental conditions.

The experimental setup is shown in figure 6(a). The imaging system consisted of eight equally spaced flexible antennas (Bahramiabarghouei *et al* 2015), identical to those used in the numerical simulations, arranged around a cylindrical breast phantom. The phantom was fabricated to match the dimensions of the numerical phantom used in the simulations, using a commercial Skin-A4–P10 skin-mimicking shell (SPEAG, Zurich, Switzerland) with a thickness of 1.5 mm, an outer radius of 73 mm, and a height of 80 mm. The shell was mounted within a custom non-conductive polyethylene terephthalate glycol-modified (PETG) enclosure, which maintained the phantom geometry and contained the liquid tissue-mimicking materials during the measurements. The phantom cavity was filled with pure glycerin, which served as the homogeneous background medium. Two hollow cylindrical inclusions fabricated from composite conductive polylactic acid (PLA), with an internal radius of 7.5 mm, a wall thickness of 2 mm, and a height of 80 mm, were embedded within the phantom. The dielectric properties of all tissue-mimicking mixtures were characterized using a calibrated Keysight N1501A slim-form open-ended coaxial dielectric probe over the frequency range from 1 to 5 GHz, corresponding to the operating bandwidth of the imaging system. The measured dielectric properties are presented in figure 6(b), and were found to be consistent with representative dielectric properties of breast tissues reported in the literature (Zastrow *et al* 2008, Cheng and Fu 2018, IT’IS Foundation 2024).

**Figure 6.**
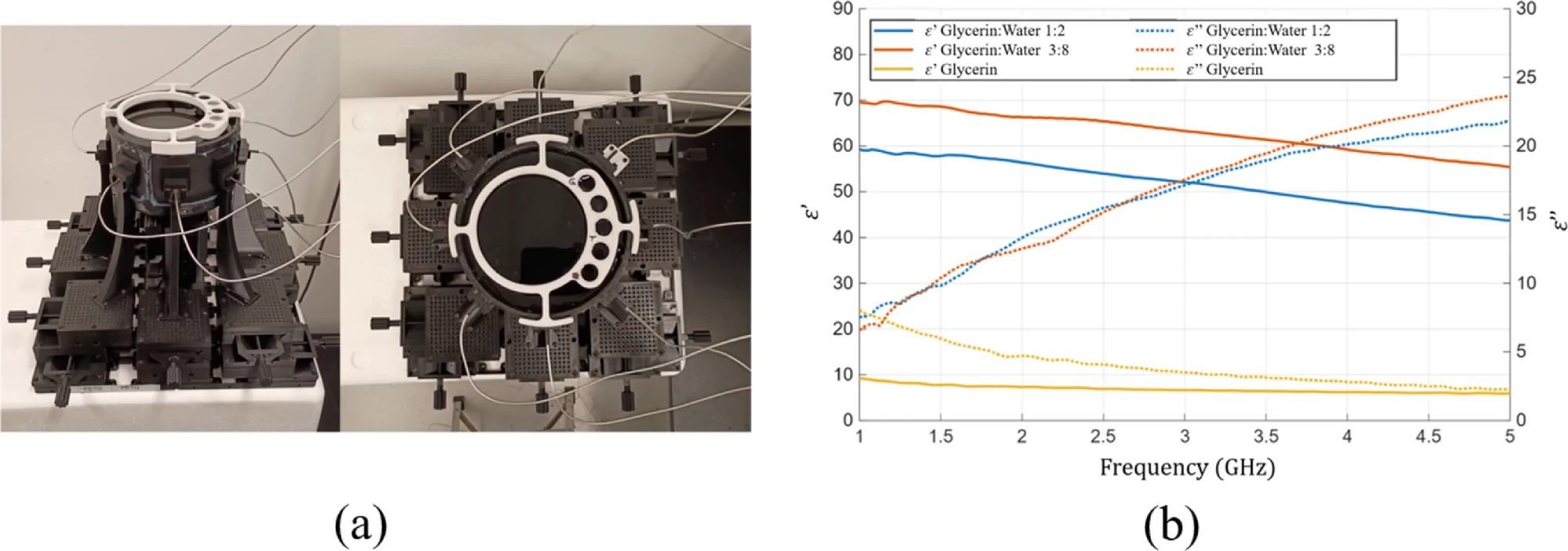
Experimental setup and dielectric characterization of the tissue-mimicking materials. (a) Photographs of the experimental imaging system showing side (left) and top (right) views of the eight-antenna measurement setup. (b) Measured frequency-dependent real (*ϵ*′, solid lines) and imaginary (*ϵ*″, dashed lines) parts of the complex relative permittivity of the glycerin background and the two glycerin-water mixtures used to emulate the tumor and glandular tissue over the 1–5 GHz frequency range.

Multi-static S-parameter measurements were acquired using a calibrated Keysight PXIe vector network analyzer over the 1–5 GHz frequency range, with 201 uniformly spaced frequency samples. During the reference measurements, both inclusion shells were filled with pure glycerin. Subsequently, the glycerin within the inclusions was replaced to generate the investigated experimental scenarios. For the first scenario, one inclusion, located at position **T** (figure 6(a)), was filled with a glycerin-water mixture having a 3:5 volume ratio to emulate a tumor, while the second inclusion (position **G**) remained filled with pure glycerin. For the second scenario, the tumor remained at position **T**, while the glycerin-filled inclusion in position **G** was replaced with a 1:2 glycerin-water mixture to emulate glandular tissue. Measurements were therefore acquired for (i) a tumor in a homogeneous background and (ii) a tumor in the presence of a glandular inclusion.

The measured data were reconstructed using the DAS and delay-multiply-and-sum (DMAS) algorithms implemented in the MERIT toolbox (O’Loughlin *et al* 2018) and subsequently evaluated using the proposed objective image quality metrics introduced in section 2.4. Unlike DAS, the DMAS algorithm is a nonlinear beamforming technique that exploits the coherence of backscattered signals by multiplying delayed signal pairs before summation. This operation enhances coherent target responses while suppressing incoherent clutter and noise, often resulting in improved image contrast and target localization.

## 3. Results

### 3.1. SNR and SMR

Figure 7 shows the SNR plotted as a function of relative permittivity for each case and for each uniformly distributed antenna array (with 6, 8, and 12 elements). In the same plot, the average and standard deviation of SNR are also shown for the symmetric and asymmetric 8-antenna arrays. As expected, increasing the number of antennas resulted in higher SNR values. The highest mean SNR was achieved by the 12-antenna configuration (25.53 dB), whereas the lowest mean SNR was observed for the 6-antenna configuration (14.60 dB). This improvement is mainly due to the larger set of independent measurements and the improved angular sampling of the scattered field provided by the denser array. Among the 8-antenna arrays, uniform and symmetric configurations exhibit higher average SNR than asymmetric ones (see table 2). The higher SNR achieved by the symmetric array configurations compared with the asymmetric configurations was confirmed to be statistically significant according to the two-sided Wilcoxon test ($p = 1.83 \times {10^{ - 5}}$). Likewise, the 12-element uniform array yielded significantly higher SNR values than the 8-element uniform array ($p = 5.96 \times {10^{ - 8}}$).

**Figure 7.**
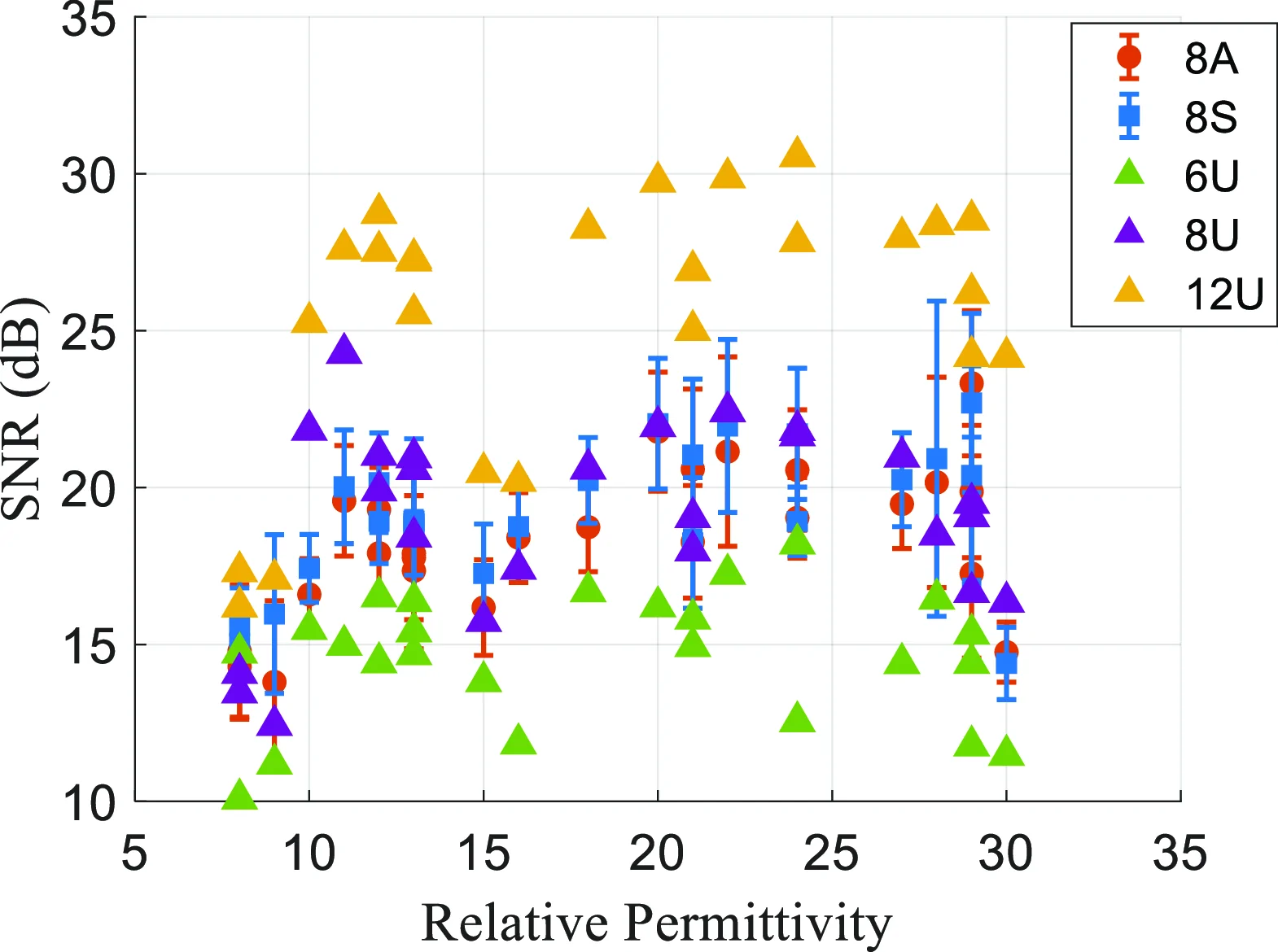
SNR as a function of the background relative permittivity for all cases. Each point represents one case. Yellow, purple, and green markers correspond to uniform circular arrays with 12 (12U), 8 (8U), and 6 (6U) antennas, respectively. Blue and orange markers indicate the average SNR ± standard deviation for symmetric (8S) and asymmetric (8A) 8-antenna configurations.

**Table 2.** Mean and standard deviation of SNR and SMR for the 8-antenna uniform, symmetric, and asymmetric array configurations.

Array Type	SNR (dB)	SMR (dB)
*Uniform*	19.07	11.30
*Symmetric*	19.11± 0.84	11.32± 0.13
*Asymmetric*	18.35± 0.89	11.17± 0.17

In terms of variation against the relative permittivity of the background, the SNR initially increases and then decreases. However, the presence of two tumors may result in noisier images and worse SNR, therefore we also present this plot excluding the two-tumor scenarios, which makes the decreasing trend with permittivity more evident, as shown in figure 8(a). Figure 8(b) shows the SMR plotted as a function of permittivity for each one-tumor case. As expected, the SMR follows a similar trend with respect to the SNR.

**Figure 8.**
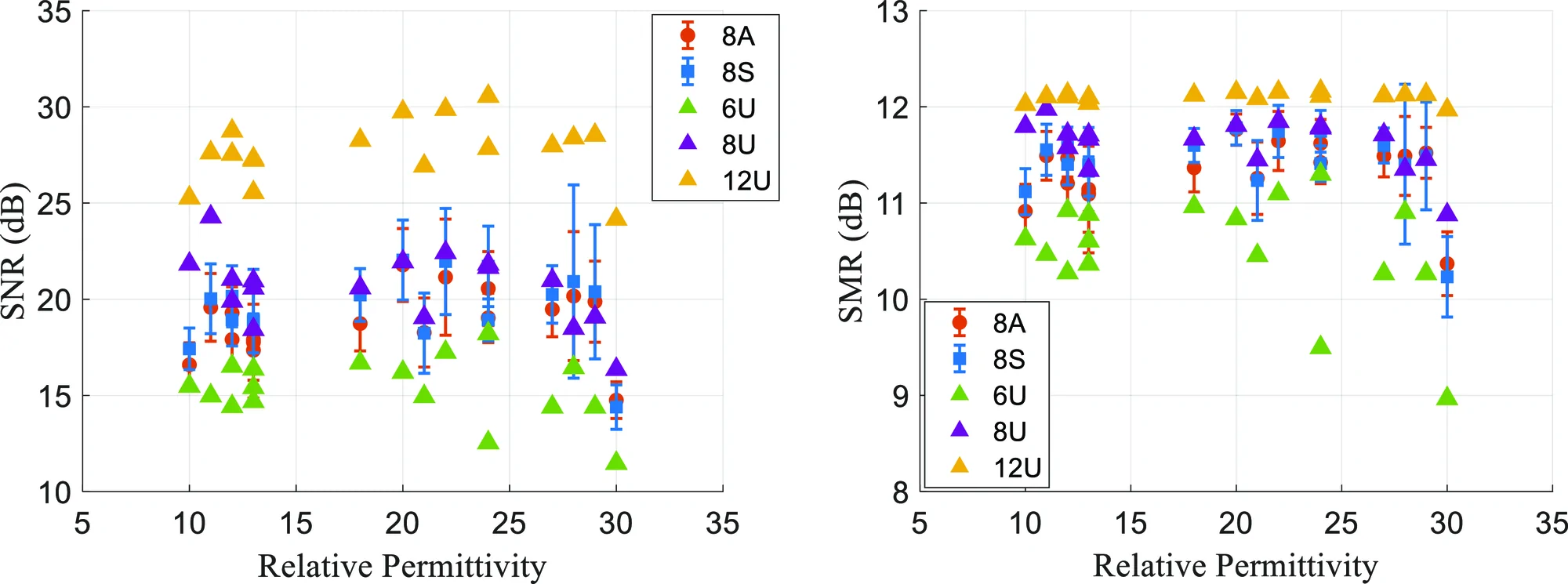
(a) SNR versus the background relative permittivity, excluding two-tumor scenarios. With single-target cases only, a clearer decreasing trend in SNR is observed at higher permittivity values due to increased loss. (b) SMR as a function of the background relative permittivity for all simulated cases. Each point represents one case. Yellow, purple, and green markers correspond to uniform circular arrays with 12 (12U), 8 (8U), and 6 (6U) antennas, respectively. Blue and orange markers indicate the average SMR ± standard deviation for symmetric (8S) and asymmetric (8A) 8-antenna configurations.

In figures 9(a) and (b), the SNR and the SMR are plotted as functions of the effective radius, respectively. The effective radius is defined as in (14):

\begin{equation*}{r_{\mathrm{eff}}} = \sqrt {\mathop \sum \nolimits_{i = 1}^n {r_i}^2{\text{ }}} \end{equation*}

**Figure 9.**
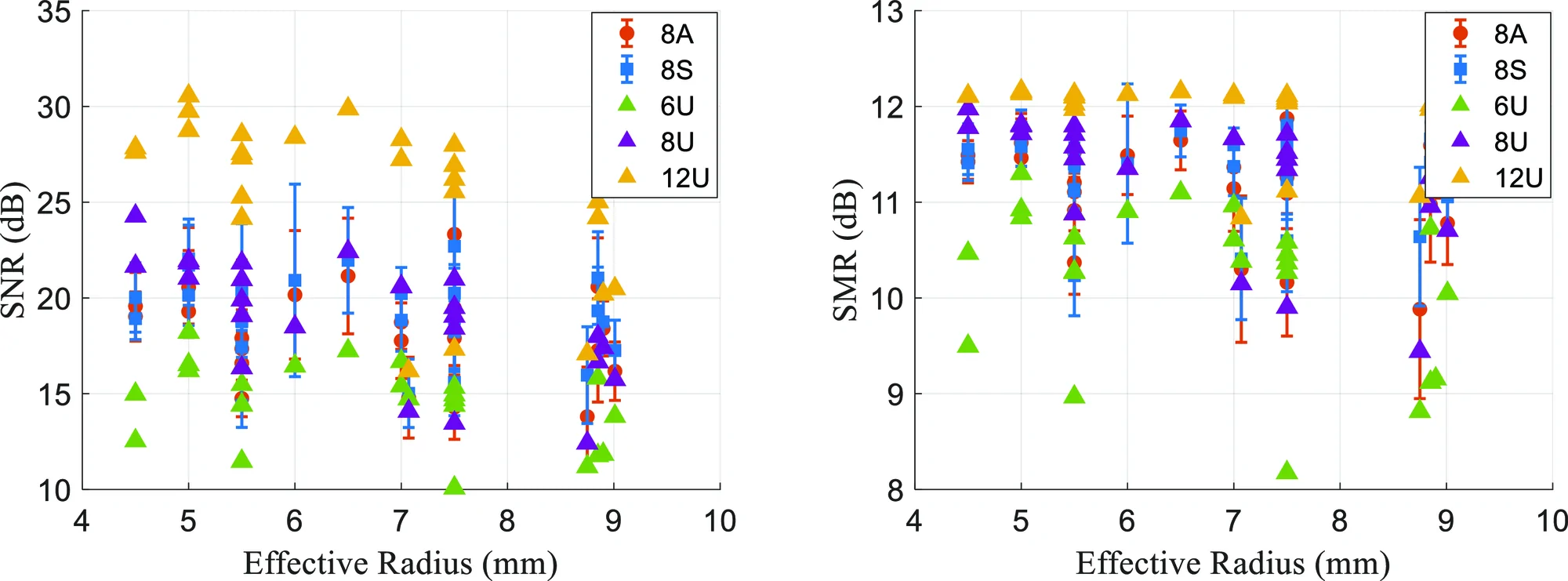
(a) SNR and (b) SMR as a function of effective radius for all simulated cases. Each point represents one case. Yellow, purple, and green markers correspond to uniform circular arrays with 12 (12U), 8 (8U), and 6 (6U) antennas, respectively. Blue and orange markers indicate the average SNR ± standard deviation for symmetric (8S) and asymmetric (8A) 8-antenna configurations.

where ${r_i}$ is the radius of the $i{\mathrm{th}}$ tumor for each image.

As shown in figures 9(a) and (b), images corresponding to two-tumor cases (7.5 mm < ${R_{{\mathrm{eff}}}}$ < 9 mm) exhibit systematically lower SNR values compared to single-tumor cases. This behavior is consistent with the representative reconstructions in figure 4, where multi-target slices (Cases 8, 10, and 18) show increased background fluctuations relative to single-target configurations. For images with one tumor only (lower values of ${R_{{\mathrm{eff}}}}$), there is not a clear trend observable across all cases. One possible explanation is that, as shown in figure 11, tumor detection performance depends not only on effective radius, but also on tumor location. The spatial position of the tumor(s) relative to the antenna array likely affects the ability to capture scattering information, which in turn influences both SNR and SMR. This may account for the observed variability and the lack of a consistent trend, particularly in more complex configurations like the two-tumor cases. Using the effective radius in two-tumor cases may introduce ambiguity, as the same value can represent different tumor sizes, affecting result interpretation. However, it remains a useful metric by providing a consistent measure of total tumor size and enabling comparison across cases and broader trend analysis.

To summarize the results, table 2 presents the mean SNR and SMR computed across all cases for the 8-antenna symmetric, asymmetric, and uniform arrays, each based on 25 images. The average and standard deviation were calculated separately for the symmetric and asymmetric configurations. These values indicate that asymmetric arrays tend to perform worse than uniform and symmetric ones.

### 3.2. Location error

Figures 10(a) and (b) show the localization error for the one-tumor cases, plotted as a function of the relative permittivity of the background and tumor radius, respectively. No clear trend is observed overall, but location error tends to be lower at low permittivities. Uniform arrays generally show lower localization error, and symmetric arrays outperform asymmetric ones in this regard. Location error appears to increase with tumor radius, possibly because larger tumors produce more diffuse scattering, making it harder to identify a clear, localized center. This can result in multiple or broader peaks, introducing ambiguity in centroid detection and shifting the estimated location.

**Figure 10.**
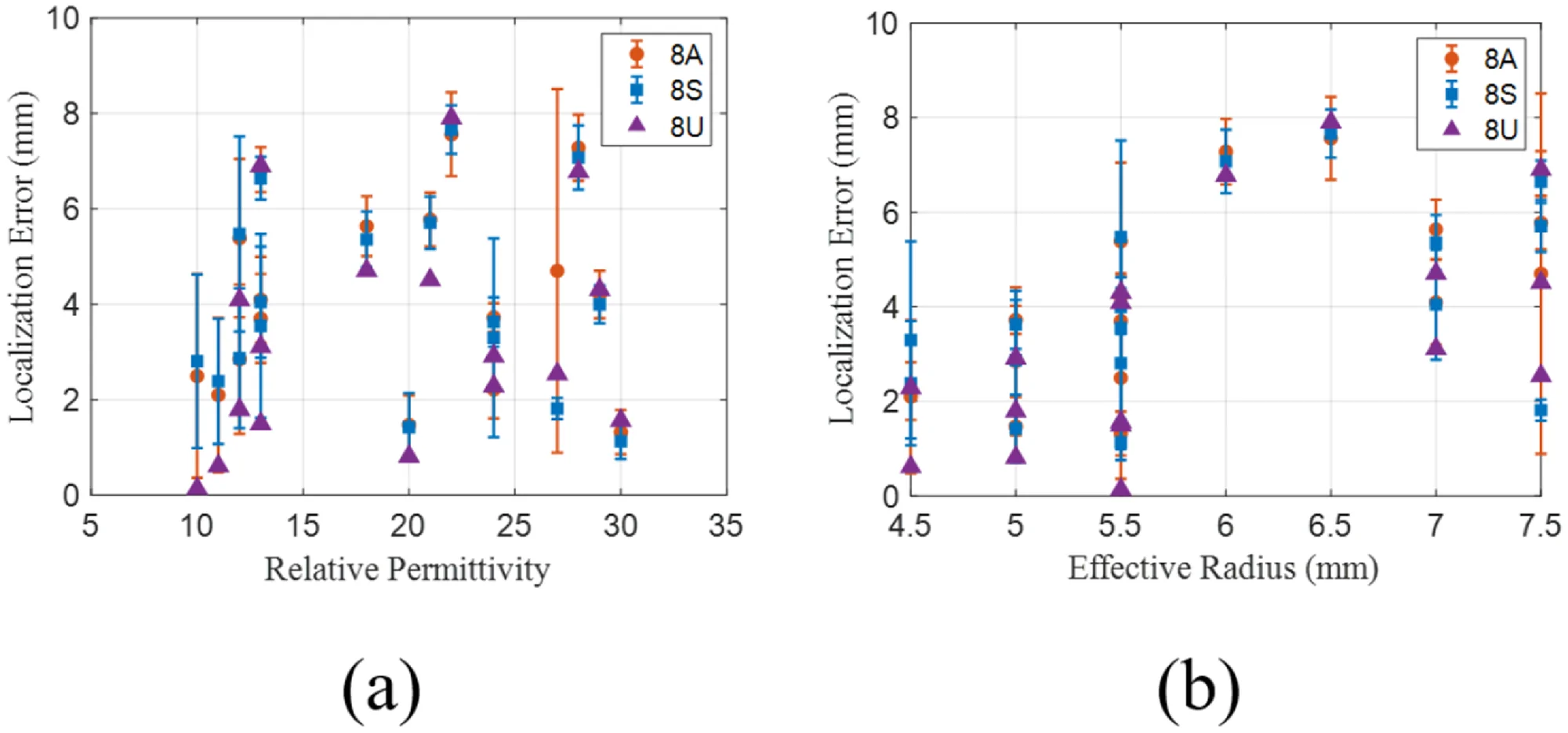
Localization error as a function of (a) background relative permittivity and (b) effective radius for one-tumor cases. Results for the uniform 8-antenna array (8U) are shown in purple. Results for the symmetric (8S, blue) and asymmetric (8A, orange) 8-antenna configurations are presented as mean ± standard deviation.

Figure 11 present 2D maps of localization error intensity for the uniform 8-antenna array configuration, where each tumor location is plotted within the breast cylinder and color-shaded to indicate the average magnitude of the error. Tumors located deep within the tissue typically exhibit low localization error, whereas the error tends to increase for tumors near the boundaries. This analysis was performed for the single-tumor case only, as the two-tumor scenarios were instead evaluated using the separation error metric.

**Figure 11.**
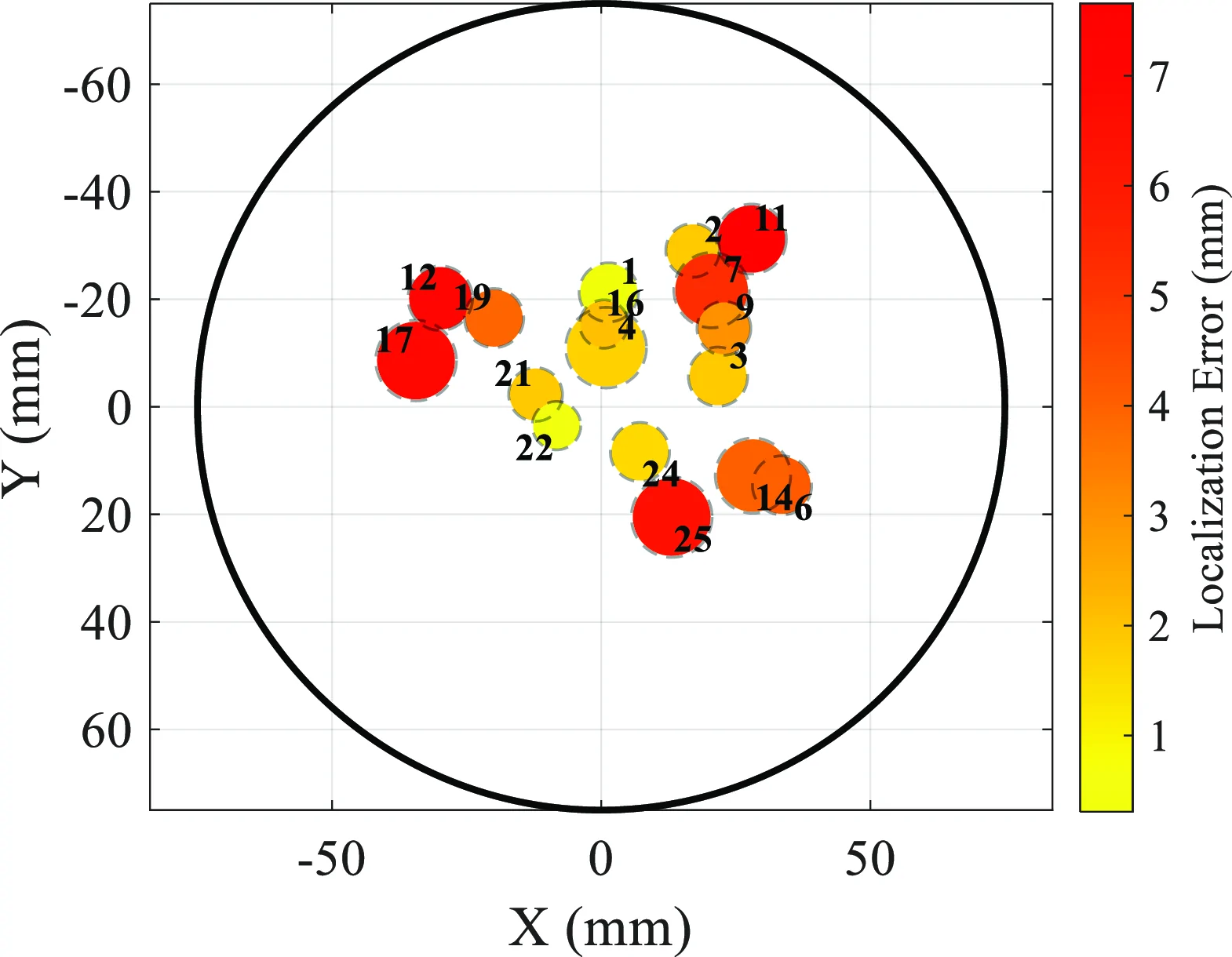
2D Coordinates of each tumor case plotted within the breast cylinder, with color shading indicating localization error magnitude in millimeters (yellow = low error, red = high error). Tumors deeper inside the tissue exhibit lower error, while those near the boundaries show increased localization uncertainty.

### 3.3. Separation error

Separation error results are summarized in table 3, showing the mean and standard deviation for each 8-antenna array configuration. The separation error is lowest for the 8-antenna uniform array. Symmetric and asymmetric configurations both produced higher values with greater variability, with the asymmetric ones showing the highest, although the difference between them was not significant in this dataset. Three subsets of array configurations (two asymmetric and one symmetric) were excluded from this analysis because, for these arrays, the smaller tumor in case 10 was not detected, preventing calculation of the separation error.

**Table 3.** Mean and standard deviation of separation error for each 8-antenna array configuration. Cases with undetected smaller tumors were excluded from this analysis.

Array type	Separation error (mm)
*Uniform*	11.01
*Symmetric*	14.37±2.62
*Asymmetric*	15.78±5.55

### 3.4. Experimental demonstration of pipeline utility

To provide an initial experimental assessment of the proposed evaluation framework, proof-of-concept measurements were performed under two representative imaging scenarios: a tumor in a homogeneous background and a tumor in the presence of a glandular inclusion embedded within the background. Figure 12 presents the DAS (figure 12(a)) and DMAS (figure 12(b)) reconstructed images for both scenarios, while the corresponding objective image quality metrics are summarized in table 4.

**Figure 12.**
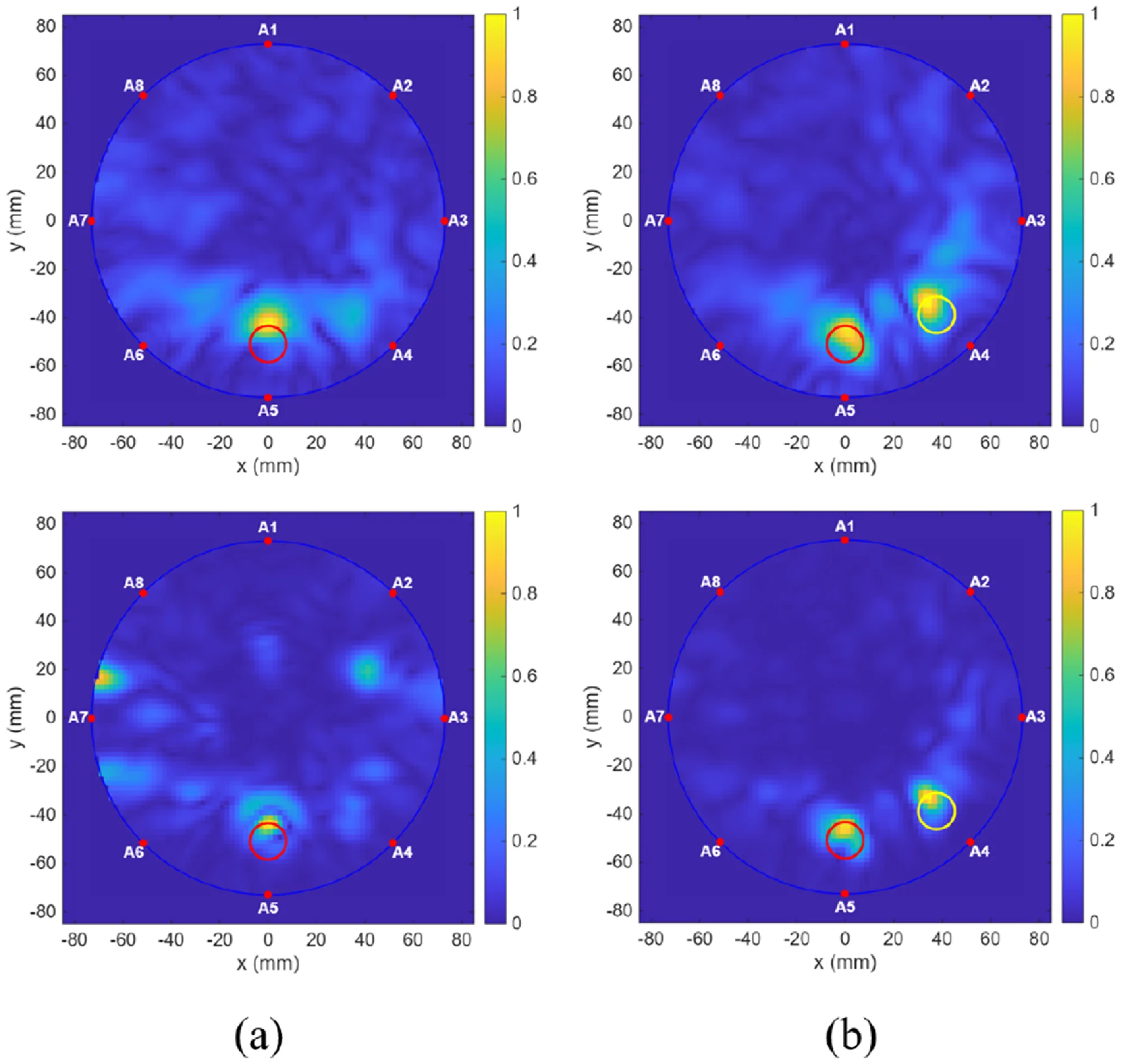
Reconstructed images obtained using (a) DAS and (b) DMAS for two experimental scenarios: (top) tumor in homogeneous background and (bottom) tumor in the presence of a glandular inclusion. Red circles indicate the actual tumor locations, yellow circles indicate the glandular inclusion locations, while red dots with labels mark the antenna positions. For both reconstruction algorithms, the input background permittivity in the MERIT toolbox was set to the measured relative permittivity of the homogeneous background medium at 3 GHz.

**Table 4.** Objective image quality metrics obtained from the experimental proof-of-concept. Localization error is reported for the tumor in a homogeneous background scenario, whereas separation error is reported for the tumor-plus-glandular-inclusion scenario.

Experimental scenario	Reconstruction algorithm	SNR (dB)	SMR (dB)	Localization error (mm)/Separation error (mm)
*Tumor in homogenous background*	DAS	13.76	10.01	12.59
	DMAS	15.92	11.90	13.32
				
*Tumor + Glandular inclusion in homogenous background*	DAS	15.51	11.74	4.90
	DMAS	21.86	13.36	2.30

For the tumor with a homogeneous background scenario, both reconstruction algorithms successfully localized the target, whereas DMAS achieved higher SNR and SMR values than DAS, indicating improved image contrast and target detectability. The localization error remained comparable for both algorithms. The second experimental scenario introduced a glandular inclusion to incorporate a degree of tissue heterogeneity into the phantom. Under this condition, both the tumor and the glandular inclusion generated visible responses in the reconstructed images. This observation is consistent with the characteristics of radar-based MWI, where the reconstructed intensity depends not only on the dielectric contrast of the scattering target but also on its position within the imaging domain and its proximity to the antenna array, both of which influence the strength of the measured scattered fields. In the present experimental configuration, the glandular inclusion was positioned approximately 4 mm closer to the nearest antenna than the tumor, which may have contributed to the comparable hotspot intensities observed in the reconstructed images. Despite the increased complexity introduced by the heterogeneous phantom, DMAS again achieved higher SNR and SMR values than DAS, together with a lower separation error.

## 4. Discussion

This study systematically evaluated how controllable factors (i.e. single-ring circular array architecture and the number of antenna elements) and uncontrollable factors (e.g. tumor size, position, tissue relative permittivity) affect MWI performance. Our simulation findings are based on DAS reconstructions; different algorithms may yield varying trends depending on their sensitivity to noise, resolution, or regularization strategies. Furthermore, the obtained results are inherently dependent on the specific antenna design adopted in this study.

Nevertheless, the proposed pipeline is conceived as a general and flexible methodology for the systematic evaluation of microwave breast imaging systems. Although the present study focuses on a single-ring, circular breast-in-contact array configuration, the methodology is not inherently restricted to this setup and can be extended to different antenna designs, array geometries, coupling conditions, and reconstruction algorithms. This flexibility is illustrated by the experimental proof-of-concept. While the simulation study employed the pipeline to compare antenna array configurations under controlled conditions, the experimental study showed that the same methodology can also be used to objectively compare alternative reconstruction approaches. The selected image quality metrics consistently captured the performance differences between DAS and DMAS, providing a reproducible basis for evaluating different design choices.

More broadly, meaningful comparisons between alternative system designs require a systematic evaluation methodology capable of isolating the contribution of individual design variables while maintaining consistency across the imaging pipeline. Similar challenges have been recognized in other computational imaging fields, particularly neuroimaging (Kiar *et al* 2020), where systematic evaluation strategies have improved reproducibility and enabled objective comparisons of alternative approaches. The proposed pipeline therefore represents a first step toward such a systematic evaluation methodology for microwave breast imaging.

For the simulation scenarios investigated in this study, both the number of antennas and the array geometry were found to influence image quality. Increasing the number of antennas improved the spatial sampling of the scattered EM fields and the angular diversity available for image reconstruction, resulting in higher image quality. Accordingly, the 12-element array achieved the highest mean SNR (25.53 dB), whereas the 6-element array yielded the lowest mean SNR (14.60 dB). Among arrays with the same number of elements, array geometry emerged as the key differentiating factor. The uniform and symmetric 8-element configurations achieved similar mean SNR values (19.07 dB and 19.11 ± 0.84 dB, respectively), both outperforming the asymmetric configuration (18.35 ± 0.89 dB). This behavior can be attributed to the more homogeneous spatial sampling and angular coverage provided by uniform and symmetric layouts. Overall, these findings indicate that increasing the number of antennas provides the greatest improvement in image quality. However, when practical constraints require fewer sensing elements, preserving a uniform or symmetric array geometry should be considered a key design principle for maintaining image quality while reducing hardware complexity, acquisition time, and system cost.

No strong performance trends were observed with respect to tumor size or tissue permittivity, though higher permittivity backgrounds showed moderately reduced SNR due to absorption. This suggests that, within the investigated range of dielectric properties, the influence of array geometry outweighs the effects of moderate variations in background tissue dielectric properties. Tumor position, however, plays a critical role: centrally located tumors were detected more accurately than peripheral ones, owing to greater angular diversity and field coverage. Specifically, centrally located tumors are illuminated and observed from a wider range of viewing angles, providing more complete scattering information for DAS reconstruction. In contrast, peripheral lesions experience reduced angular coverage and weaker effective sampling, making accurate localization and lesion separation more challenging. This reflects a limitation of fixed circular arrays, where both field strength and sampling density decrease toward the periphery. Antenna type may further influence these effects, and the pipeline developed here can be adapted to compare arrays using different antenna types.

Two-target scenarios proved especially challenging, producing noisier images and complicating lesion characterization. The increased complexity arises from the overlap of the scattered fields generated by neighboring lesions, making it more difficult for DAS reconstruction to distinguish closely spaced targets and increasing the likelihood of image artifacts. This highlights the need for stronger metrics to assess lesion separation and multi-target resolution. While the latter has been recognized as a key metric, it lacks established benchmarks in breast microwave sensing datasets (Reimer and Pistorius 2023). For this reason, as future work, we plan to integrate this measure into our evaluation pipeline to better quantify resolving capability in complex cases. Furthermore, the present analysis does not explicitly include a shape-based metric. A dedicated shape fidelity metric is expected to be highly relevant in anatomically realistic scenarios and will be addressed in future studies.

Another important consideration is that, although the objective image quality metrics adopted in this work are among the most widely used in microwave breast imaging, no consensus currently exists regarding their mathematical definition and implementation (Reimer and Pistorius 2023). Hence, the same metric may be quantified differently across studies, making direct quantitative comparisons potentially misleading and resulting in inconsistent interpretations of imaging performance. Establishing standardized metric definitions and evaluation protocols therefore represents an important direction for future research.

## 5. Conclusion

In current clinical studies, variability in system design configurations hinders comparison across studies. Therefore, a controlled analysis framework is essential to identify effective design choices before clinical translation. Although this study does not directly address complex clinical scenarios, it represents a necessary methodological step by providing a systematic and reproducible framework for quantitatively evaluating the influence of controllable and uncontrollable design factors on microwave breast imaging performance. Within the scenarios investigated, antenna array geometry was identified as a key determinant of image quality, with symmetric and uniform arrays demonstrating an effective balance between imaging accuracy and system complexity. More broadly, the proposed framework enables objective and reproducible comparisons of alternative system designs using consistent evaluation criteria, supporting the development of optimized, cost-effective, and scalable microwave breast imaging systems. Building on this proposed framework, future work will extend the analysis to fully 3D, anatomically realistic breast models incorporating heterogeneous tissue distributions, patient-specific anatomies, and practical hardware and measurement constraints, providing a foundation for subsequent clinical validation.

## Data Availability

The data cannot be made publicly available upon publication because no suitable repository exists for hosting data in this field of study. The data that support the findings of this study are available upon reasonable request from the authors.
